# How to Prevent the Warping of Gold Plates

**Published:** 1850-01

**Authors:** A. C. Castle

**Affiliations:** New York


From the Dental News Letter.

### HOW TO PREVENT THE WARPING OF GOLD PLATES.

Several articles have appeared in the Dental News Letter, and several other periodicals devoted to subjects upon dental science, regarding the twisting or warping of gold plates, intended as the basis for artificial teeth, by the action of heat upon them whilst soldering the teeth to the plate.

The articles referred to, have been edifying, instructive and amusing. Instructive from the important facts which have been collected, and the amount of unwritten and unrecorded knowledge which has been constantly increasing, and which, by the liberal and enlarged views ever observed on the well-filled pages of the Dental News, has gradually drawn the dental experience from their hiding places, to the great benefit of the whole dental profession. A few years since, it was a common occurrence, at least I experienced it to be so, that when inquiring of a professional "brother," in what manner he prevented his "suction" plates from warping, the invariable answer was, "My plates never do warp." Hence, the inquirer was not only led astray, but such statements at once led him into the conviction or supposition, that he either did not rightly understad his business, or otherwise, was a mechanical bungler. His pride, henceforward, would prevent him acknowledging such accidents as the warping of gold plates in his manipulations. Many thanks are due to dental periodicals, and those dentists with *cacœthes scribendi.* By these the dental mechanic has been from time to time furnished with the details of the complaints, confessions and remarks upon these untoward accidents in the practice of several of the members of the dental profession, which have exacted enquiries, how such results, from the application of heat to the metal, were brought about or produced, and by what method such vexing perplexities could be obviated or overcome. It is not necessary here, to review the remarks of the several writers upon the various supposed causes, and their various modes of prevention; for such unfortunate terminations to all the skill, time and patience, devoted upon their labor; sufficient will it be, in your limited space, to show in as succinct a manner as possible, how such disasters may be prevented; nay, defied from the immediate commencement of taking in hand, the formation of the plate to the model of the palatine arch of the mouth, to the final blast of the blowpipe, to complete the workmanship of a complete set of teeth.

Some years since, I was not well enough acquainted with the metallurgy pertaining to the peculiarities of dentistical mechanism, to protect myself against the frequent recurrence in my laboratory of the accidents referred to. It was my fortune, at that period, to be engaged upon a complete piece of dental mechanism, comprising the upper and lower sets of teeth for the mouth of an eminent metallist and long established practical jeweler; stating to him these difficulties which frequently perplexed me. He at once gave me the so much desired information, both to myself, and, as I find, to my professional brethren also. Years of experience, trials and attention to dental metallurgy, have confirmed the merits of the information which I received, and my experiments have exhibited the important fact, that the apparent difficulty of preventing the warping of gold "suction" plates for atmospheric pressure cases, can easily and most successfully be obviated. At present, it is impossible for me to clearly demonstrate, or even offer an opinion that will satisfactorily explain or be acceptable, regarding the *modus operandi* of the action of the agents I shall mention, upon the gold. All I can offer for the benefit of the readers of the Dental News, are the simple facts :— The gold being prepared for use, always bear in mind, that the gold as prepared and *alloyed* by Mr. A. Jones, is about the best quality to test their truth. If too much copper or *bad "filings"* be alloyed with the gold, warping of the plates is most certain to follow the application of heat. Also, using solder with too much copper, has the effect of contracting itself or drawing inward upon the face or line of solder, the gold basis upon which the teeth are fitted. The gold basis previous to being struck up or "swedged"(?) upon the dental die, should be heated to a dull red heat, and whilst in this state thrown into common molasses; after which, it is to be heated again in a similar manner, and cast into the diluted sulphuric acid ("pickle,") and then struck up to the dental die; being properly fitted, repeat the process with the "pickle," and it is now fully prepared with little hazzard of any warping from the effects of re-application of heat, unless the heat applied is carried to such an extent as to "sweat" its surface. To make *certainty doubly sure*, the following precautions will repay the trouble bestowed. Presuming the teeth to be ground properly, fitted, and in close apposition with the gold basis, and encased with the usual protection of sand and Plaster of Paris. The heat should be applied to the base of the casing on the palitine gum side of the gold plate; and not to the lingual or teeth surface of the exposed gold and teeth linings. The observation of this feature in the operation of soldering is of great importance. The plaster casing thus first contracting at the base binds the teeth close to the external or gum portion of the plate. The teeth, also, become heated with less hazzard of their cracking. By heating the case first, on the lingual or teeth surface, the plaster is apt to shrink and contract its circle, so that the cutting lines or edges of the teeth are drawn inwards, and the superior lines or gum edges are drawn off and away from the gold plate, so that all the time and trouble devoted to a close fitting are lost to the operator, and the beauty of the work spoiled. This will, also, occur by the use of common gold solder. The copper, as I have before stated, contracting more than the gold, when it cools, draws the plate upwards and inwards towards the internal median line of the central incisors. It is, therefore, of importance, that the dental mechanic should not use solders at different times of various qualities. The larger the case, the more difficult will it be to manage a strange solder; be it weak or strong, or, in other words, of a high or low carat in quality. This difficulty is often met with, in repairings done to atmospheric pressure cases made by other hands. A superior or inferior quality of solder being applied, causing the plate to warp from the above stated causes; and the dentist repairing the case has to submit to the remarks without being able to assign a reason for his apparent want of skill. I have hurriedly penned the above few remarks, with what benefit to your readers I leave them to acknowledge, after their experiments shall have tested its superiority.

Very respectfully,

A. C. CASTLE, M. D.

New York, December 10, 1849.

